# Supplementary material for: Regulation of Let-7a-5p and miR-199a-5p Expression by Akt1 Modulates Prostate Cancer Epithelial-to-Mesenchymal Transition via the Transforming Growth Factor-β Pathway
Source: Cancers (Basel). 2022 Mar 23;14(7):1625. doi: 10.3390/cancers14071625 (PMC8996869; doi:10.3390/cancers14071625)

Alk5

DU145

Figure 9a

1

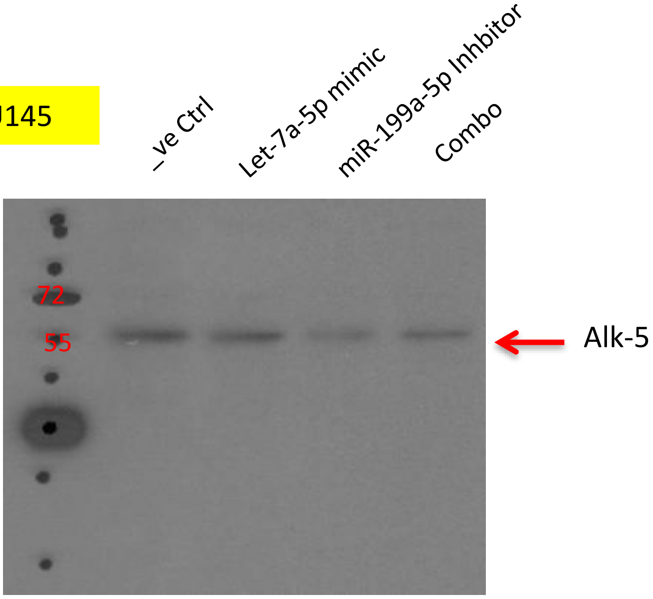

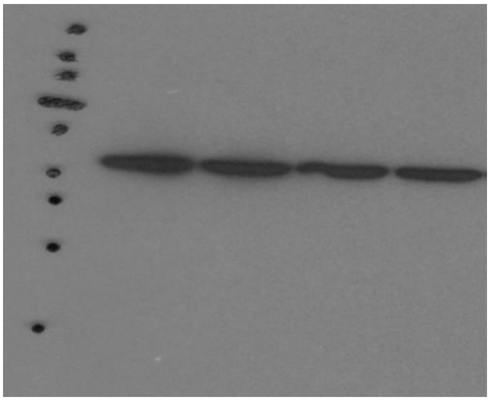

2

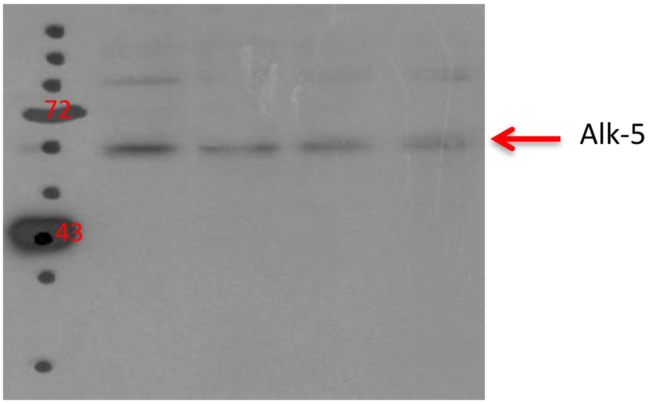

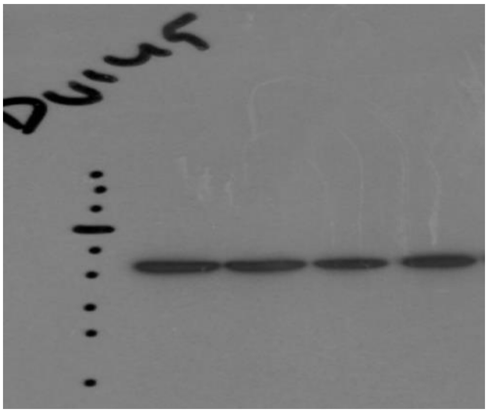

Images used in the manuscript

3

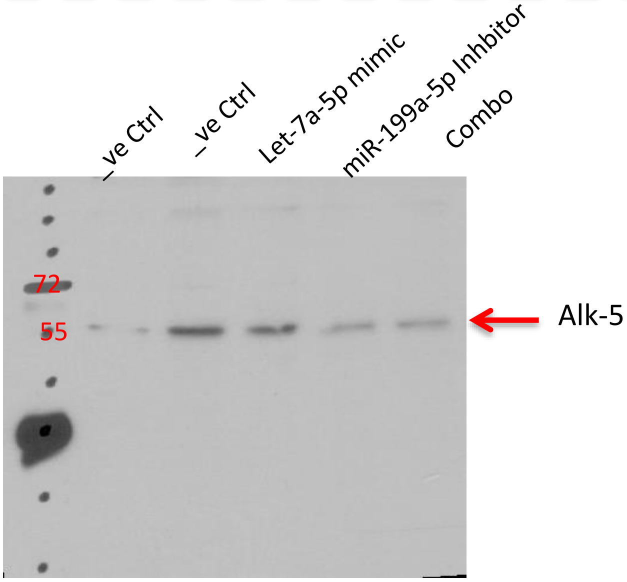

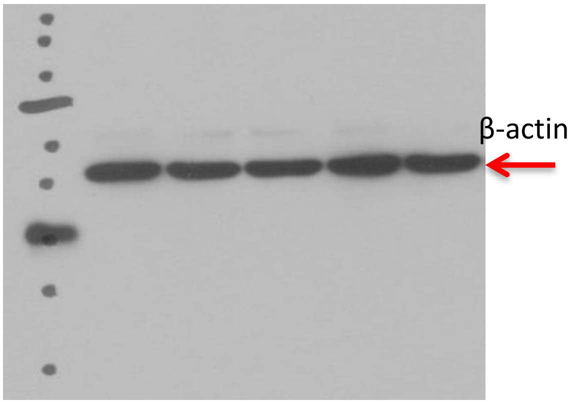

Alk5

PC3

1

Figure 10a

Images used in the manuscript

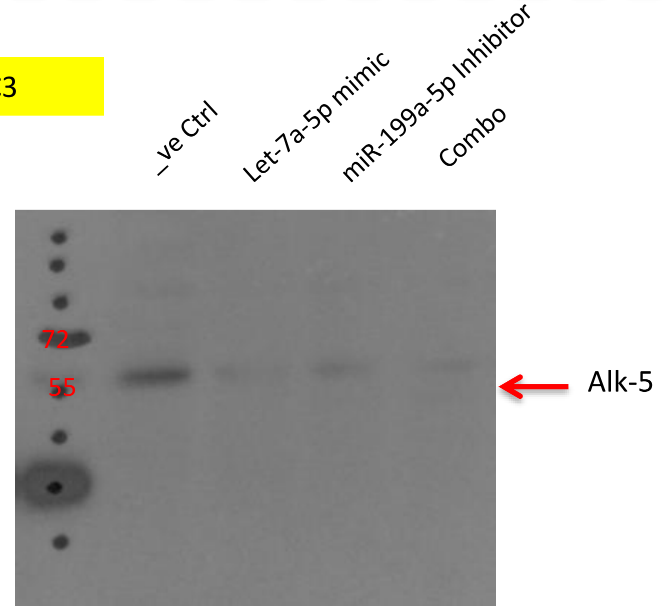

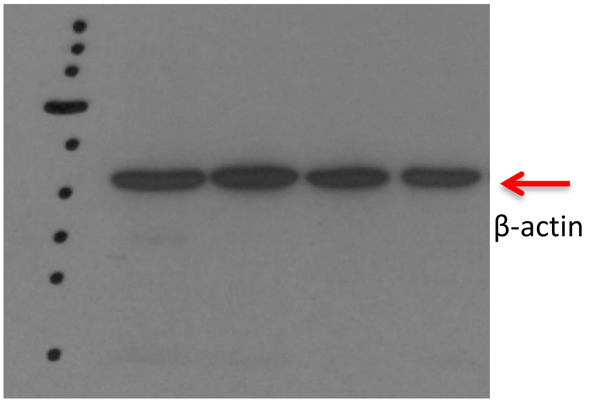

2

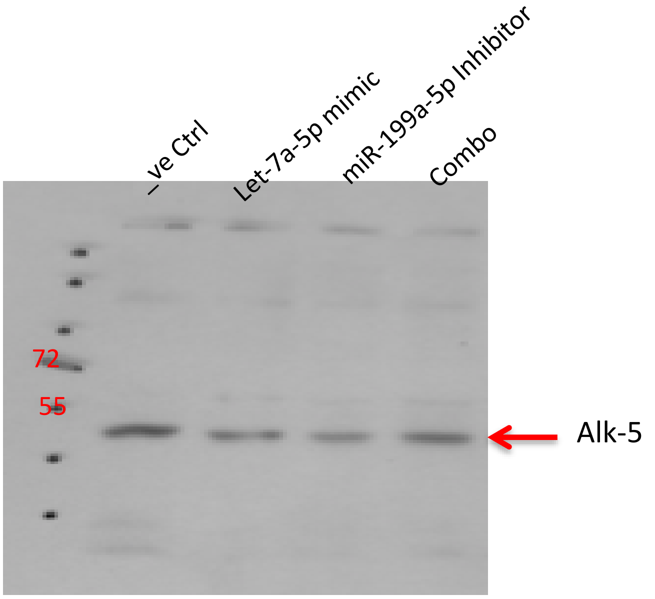

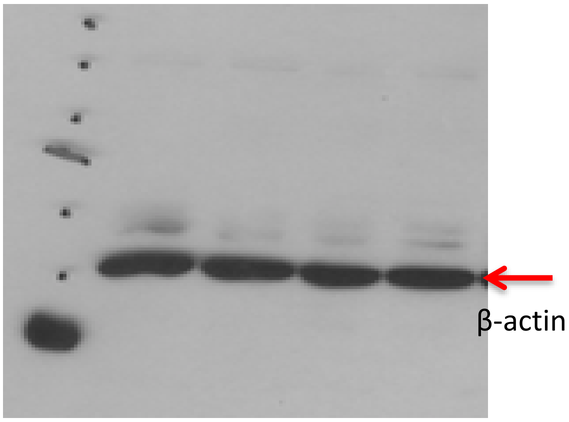

3

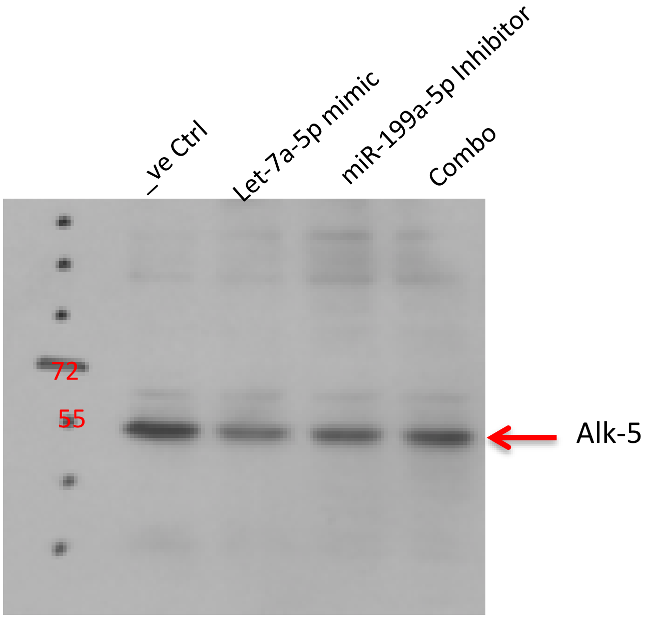

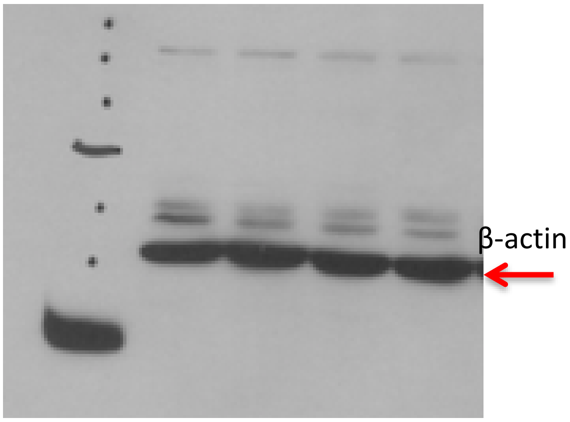

DU145

Figure 9b

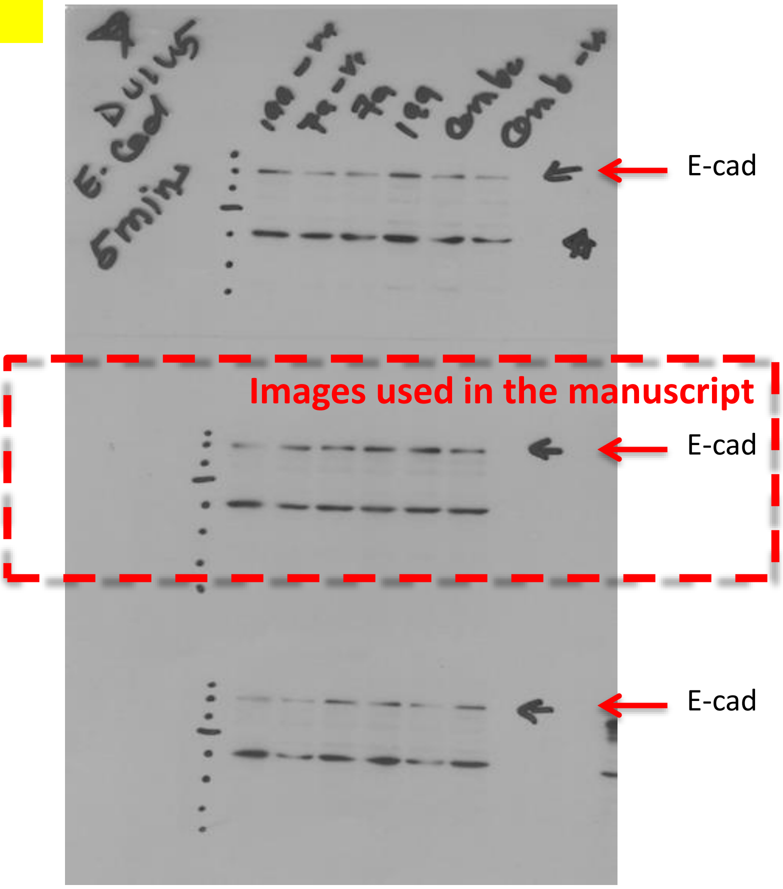

E-cad

Images used in the manuscript

E-cad

E-cad

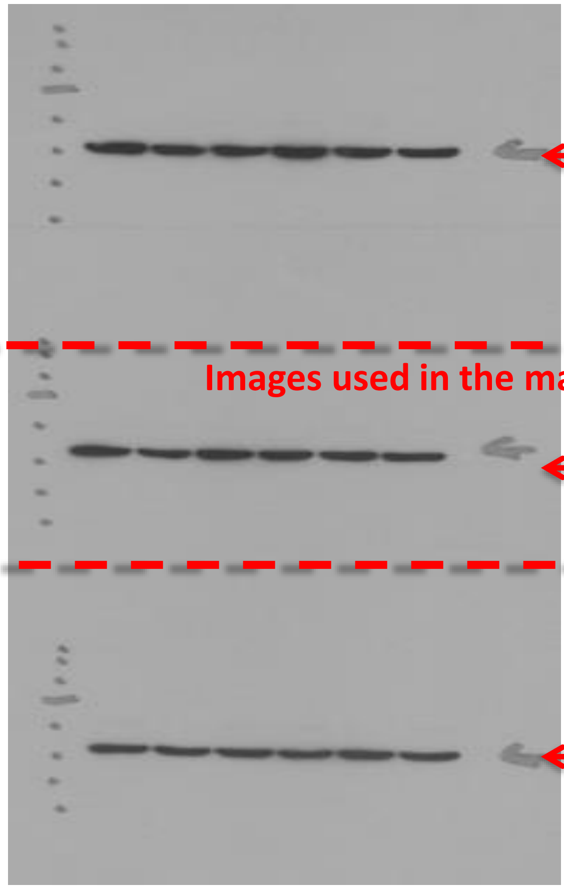

B-actin

Images used in the manuscript

B-actin

B-actin

PC3

Figure 10b

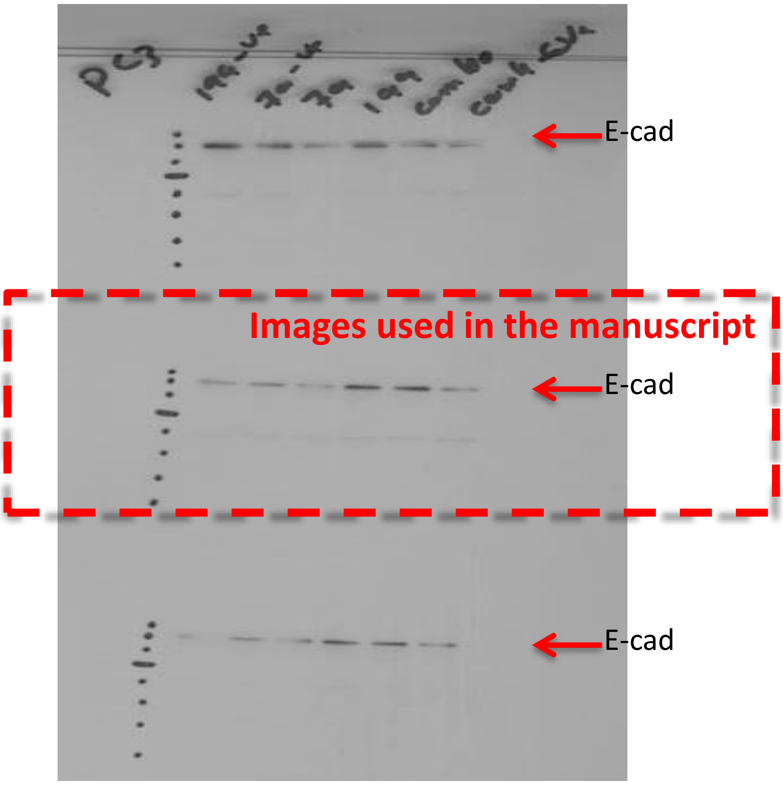

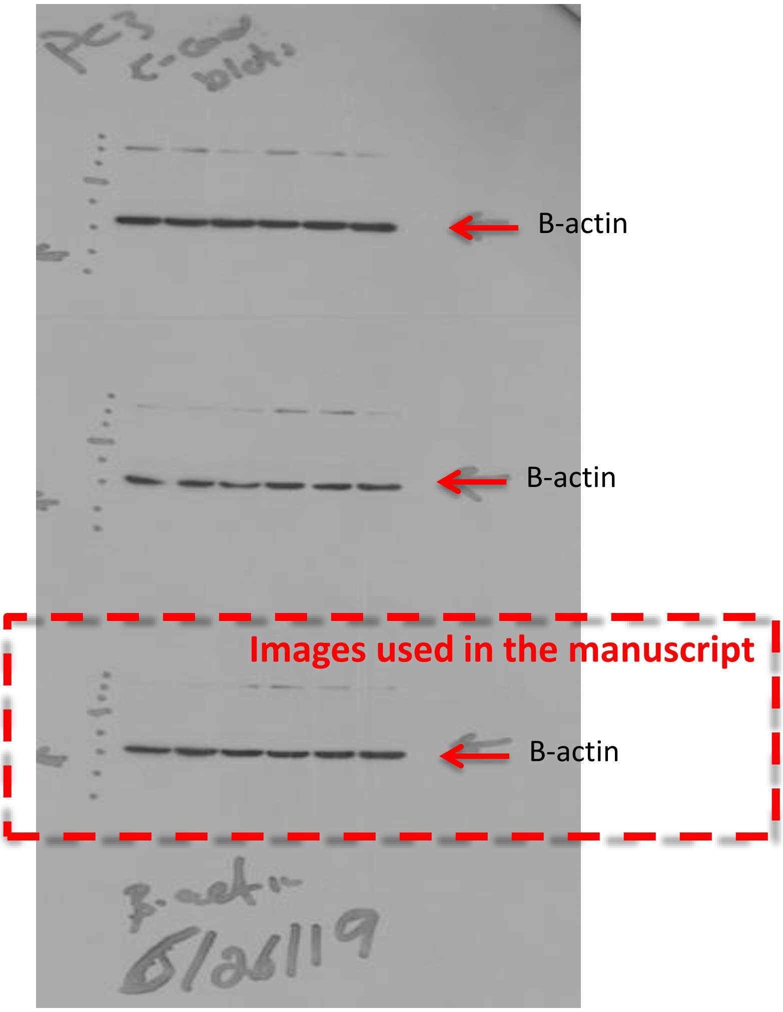

Supplement: Supplementary file 1 [file cancers-14-01625-s001.zip › cancers-1585282-supplementary-proof-send to xml/cancers-1585282-supplementary-original images.pdf]
