# Supplementary material for: Regulation of Let-7a-5p and miR-199a-5p Expression by Akt1 Modulates Prostate Cancer Epithelial-to-Mesenchymal Transition via the Transforming Growth Factor-β Pathway
Source: Cancers (Basel). 2022 Mar 23;14(7):1625. doi: 10.3390/cancers14071625 (PMC8996869; doi:10.3390/cancers14071625)
Supplement: Supplementary file 1 [file cancers-14-01625-s001.zip › cancers-1585282-supplementary-proof-send to xml/cancers-1585282-supplementary-proof.pdf]

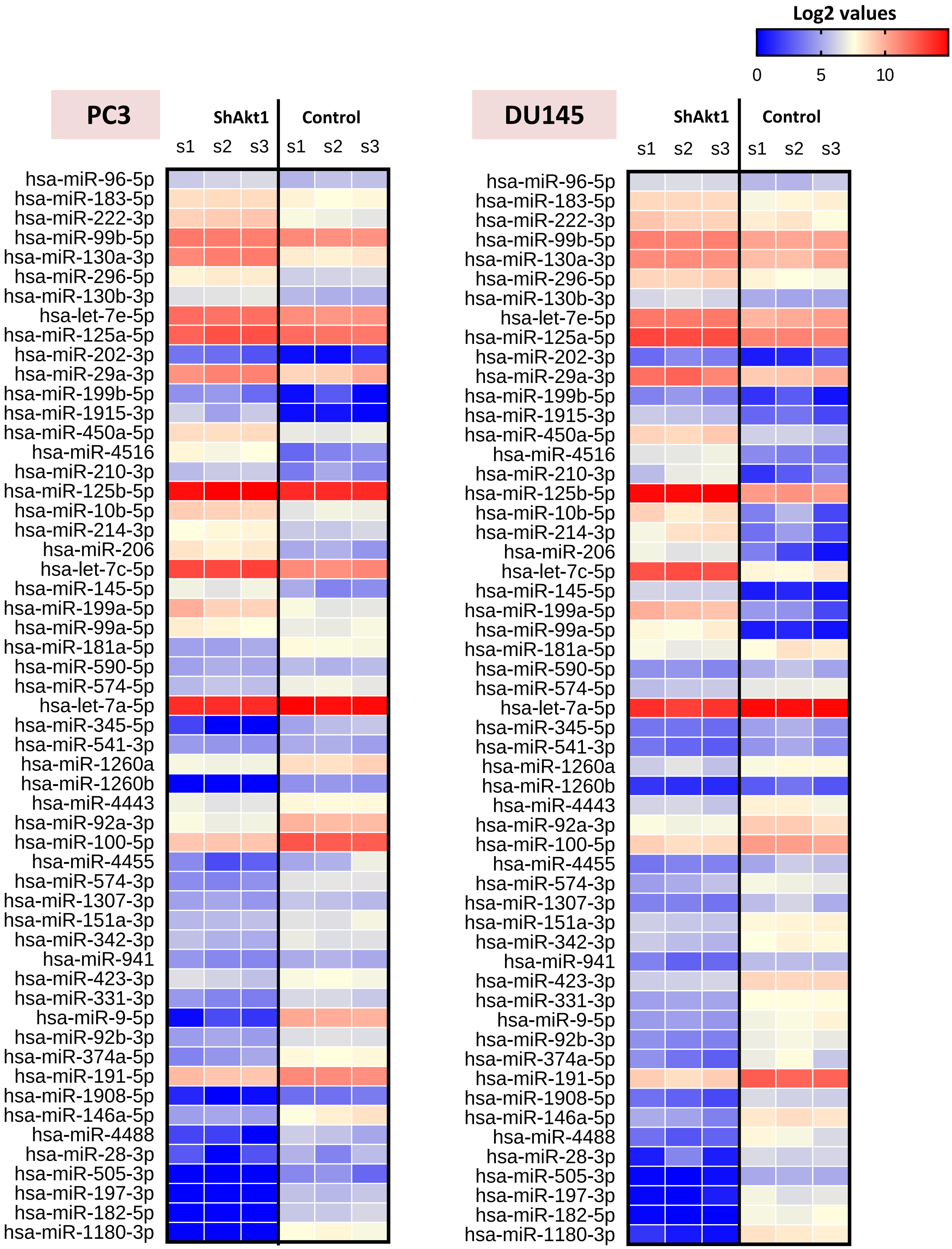

Supplemental Figure S1: Heatmaps of selective microRNAs that are modulated in PC3 and DU145 PCa cells upon shRNA-mediated Akt1 knockdown compared to shControl.

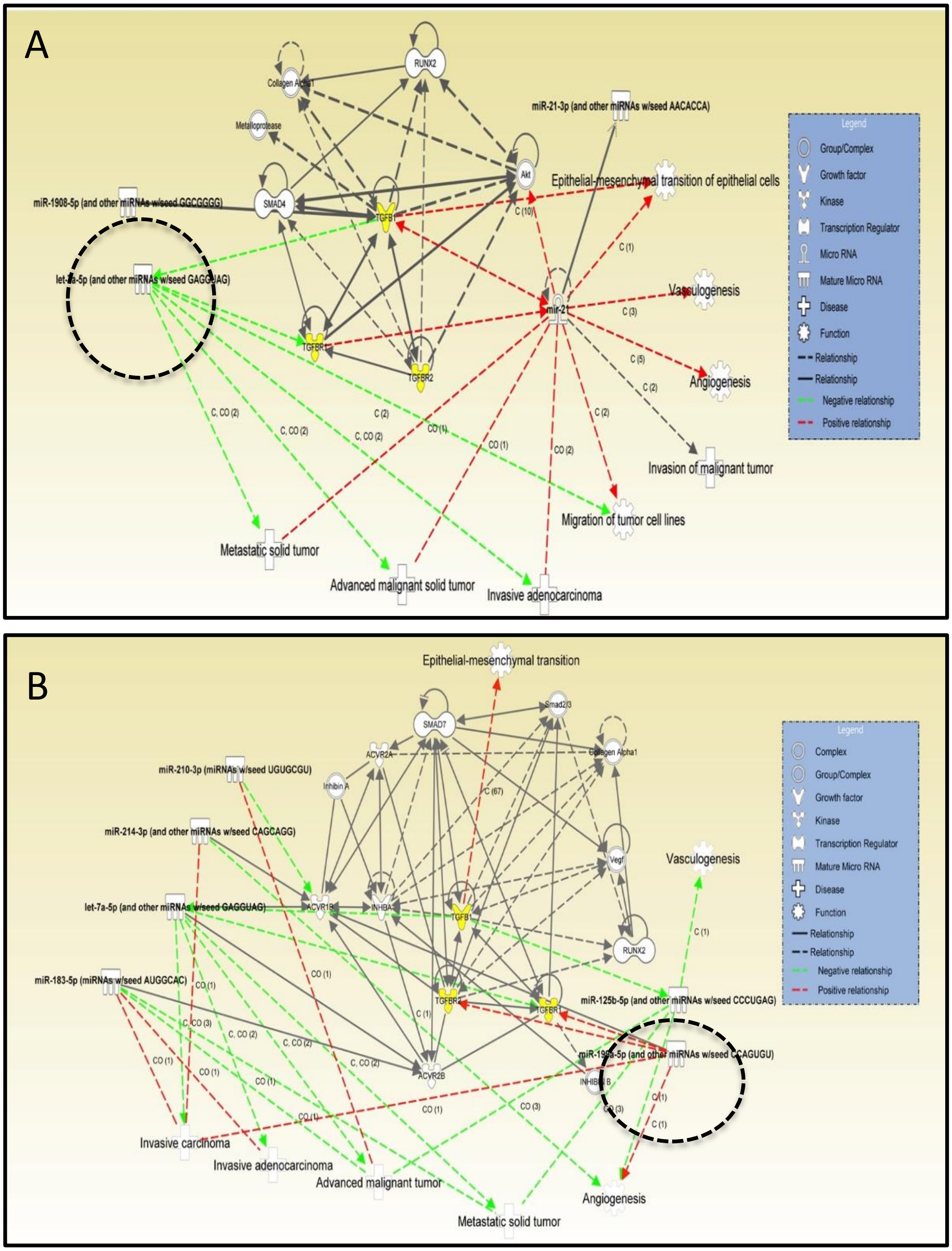

Supplemental Figure S2: Ingenuity pathway analysis of upregulated and downregulated miRNAs. **(A)** Ingenuity pathway analysis for all downregulated miRNAs mapped to PI3Kinase-Akt, TGFβ and Wnt signaling pathways show that out of the 35 miRNAs, 5 miRNAs only were linked to TGFβ pathway among which Let-7a-5p only showed a negative relationship to TGFβ1 and TGFβ-R1 expression, cancer invasion and metastasis. **(B)** Ingenuity pathway analysis for all upregulated miRNAs mapped to PI3Kinase-Akt, TGFβ and Wnt signaling pathways show that out of the 26 miRNAs, 3 miRNAs only were linked to TGFβ pathway among which miR-199a-5p only showed a positive relationship to TGFβ-R1/R2 expression, cancer invasion, and metastasis.

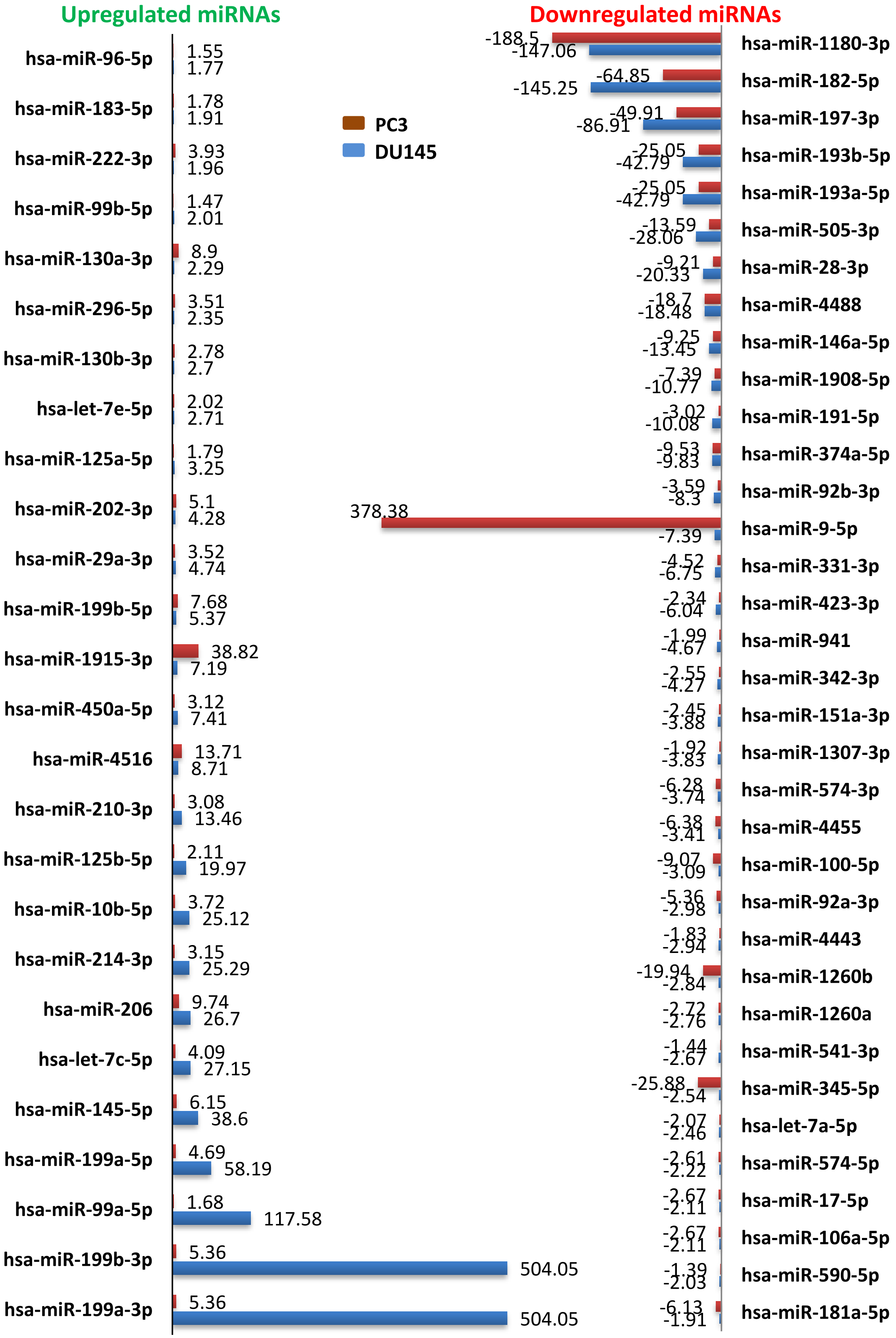

Supplemental Figure S3: Histograms showing microRNAs that are up- and downregulated in PC3 and DU145 PCa cells upon shRNA-mediated Akt1 knockdown compared to shControl.

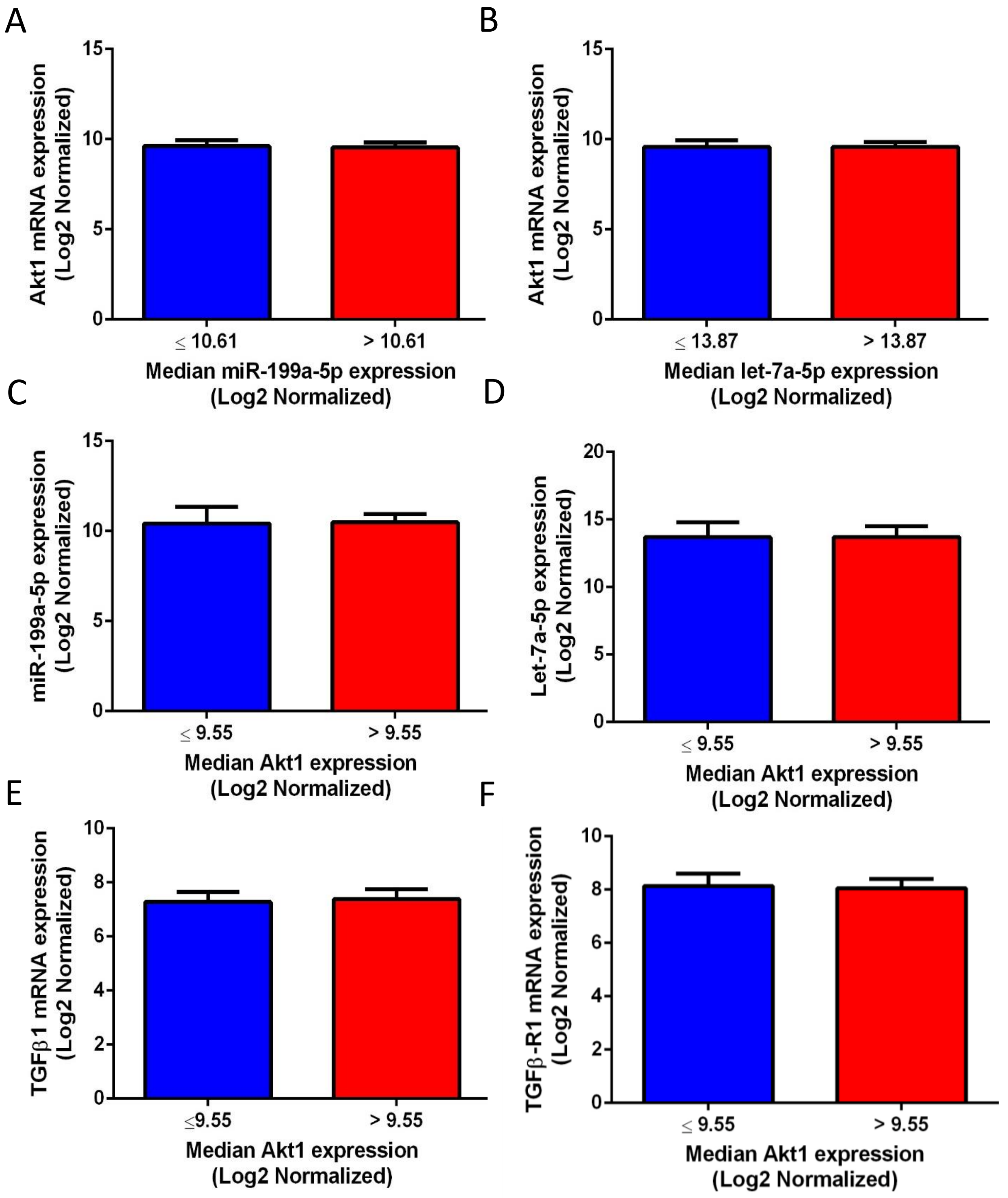

Supplemental Figure S4: Expression of Akt1, TGFβ1 and TGFβ-R1 in relation to miR-199a-5p and let-7a-5p in PCa tissues. The mRNA levels of Akt1 were investigated in PCa tissue using the median expression of each miR-199a-5p and let-7a-5p as a cutoff point to consider high vs. low expression of each miRNA. The mRNA level of Akt1 does not significantly correlate with changes in miR-199a-5p (A) and let-7a-5p (B). With respect to the impact of Akt1 on miRNAs and mRNA of TGF**β**1 and TGF**β**-R1 and after considering median Akt1 expression as the cutoff point, no significant changes have also been observed in the miRNAs (despite the results shown in Fig 7A), TGF**β**1 and TGF**β**-R1 mRNA levels. Data is presented as mean ± SD.

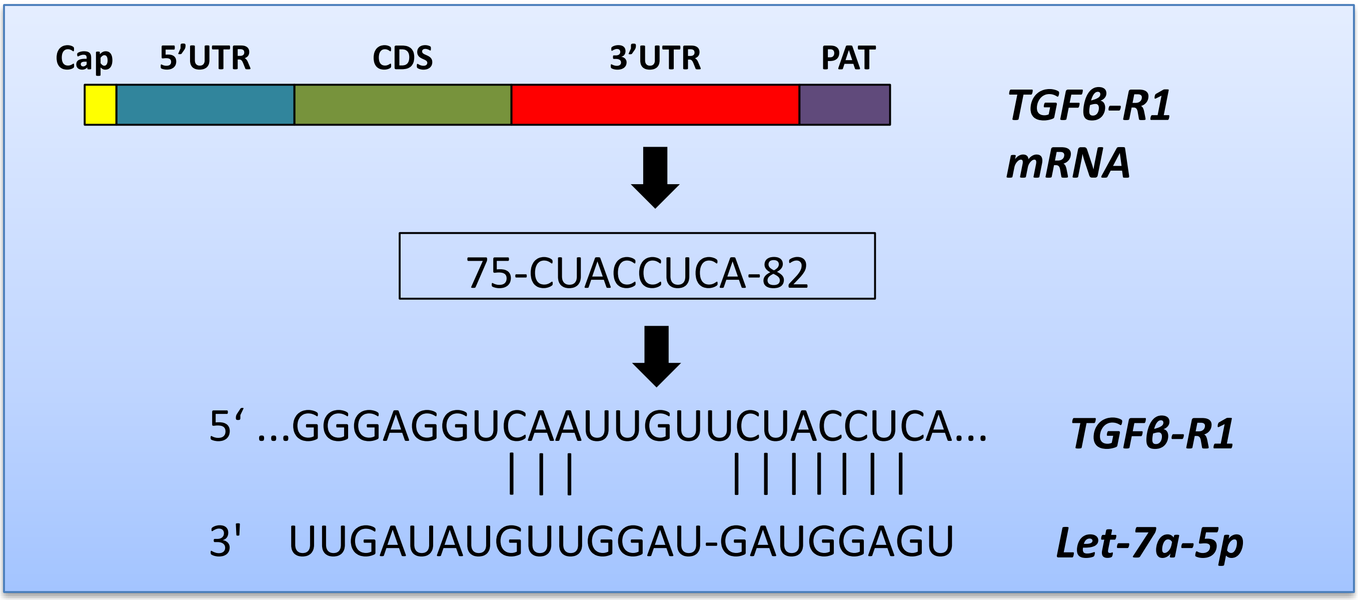

Supplemental Figure S5: Targetscan® predicted TGFβ-R1 a target of let-7a-5p, but not miR-199a-5p. TGFβ-R1 is a target of let-7a-5p based on Targetscan and downregulation of let-7a-5p accompanied by Akt1 inhibition is expected to increase TGFβ-R1 expression. TGFβ-R1 does not indicate a target site for miR-199a-5p, indicating that miR-199a-5p might inhibit a gene that negatively regulates TGFβ-R1.

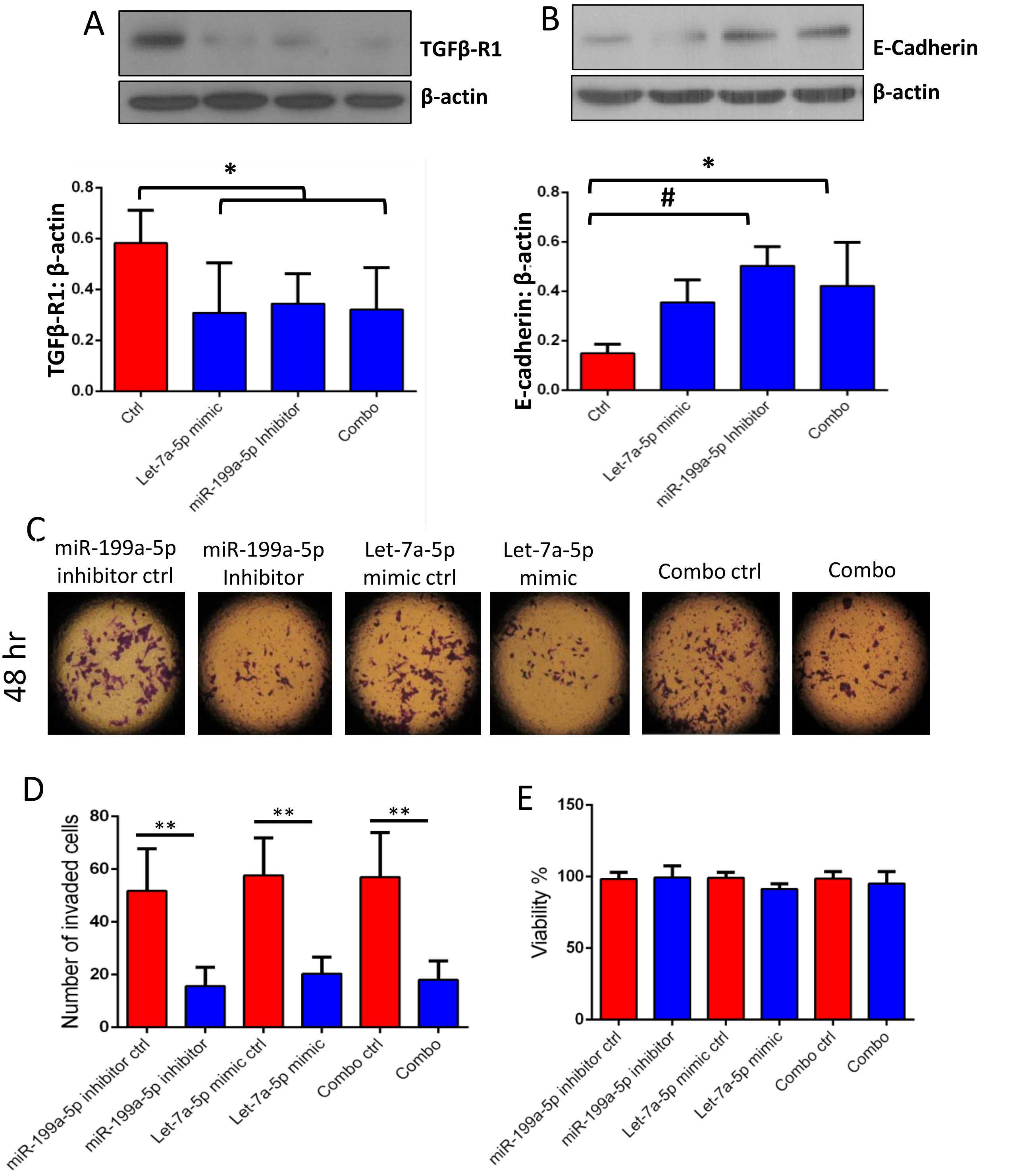

Supplemental Figure S6: Inhibition of miR-199a-5p and activation of let-7a-5p inhibited TGFβ-R1 expression, EMT and PC3 cell invasion. (A) Representative Western blot images and bar graph showing band densitometry analysis of miR-199a-5p inhibitor (25nM) and/or let-7a-5p mimic (10nM) effects in PC3 cells on TGFβ-R1 and E-Cadherin expression (n=3). (B) The use of miR-199a-5p inhibitor (25nM) and/or let-7a-5p mimic (10nM) in PC3 cells enhanced E-Cadherin expression (n=3). (C-D) miR-199a-5p inhibition and/or let-7a-5p activation in PC3 cells resulted in impaired cell migration (n=3). (E) Neither miR-199a-5p inhibition nor let-7a-5p activation in PC3 cells had any significant effect on cell viability (n=3). *($P < 0.05$); **($P < 0.01$); #($P < 0.001$); unpaired Student t-test for two groups analysis (GraphPad Prism 6.01). Data are presented as mean ± SD.

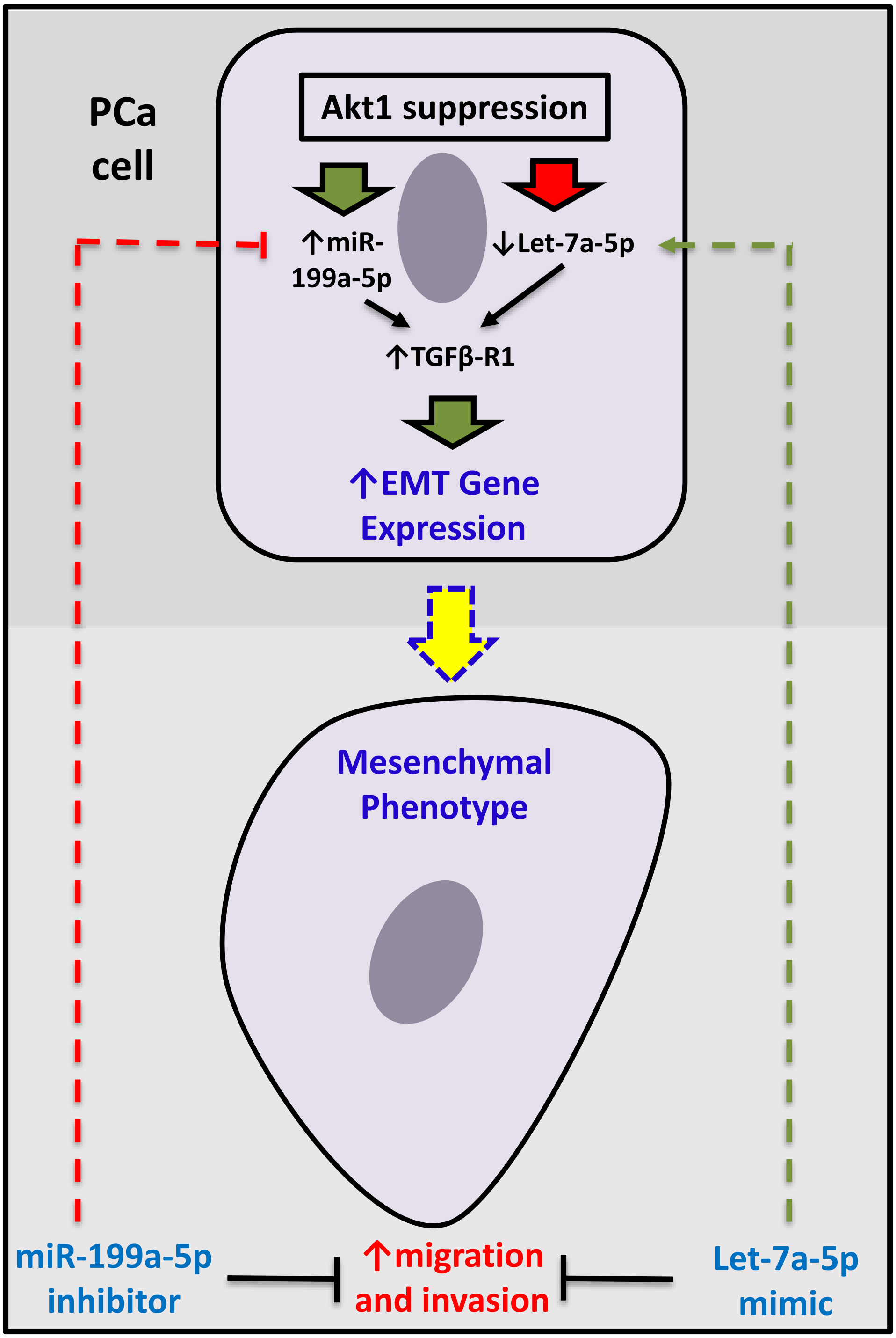

Supplemental Figure S7: Working model of promotion of EMT, cell motility and invasion in PCa cells by Akt1 suppression, which activates expression of pro-EMT miR-199a-5p and inhibits expression of anti-EMT let-7a-5p, together promoting TGFβ-R1 expression. The study identifies changes in miR-199a-5p and let-7a-5p as biomarkers for metastatic PCa and suggests pharmacologically targeting them will have therapeutic benefits for PCa.
